# The distinctiveness of human aging I: Anthropological and developmental foundations

**DOI:** 10.1007/s00391-026-02608-8

**Published:** 2026-07-28

**Authors:** Dale Dannefer, Jessica Kelley, Olugbenga Okunade

**Affiliations:** Department of Sociology, Case Western Reserve University, 10900 Euclid Avenue, 44106 Cleveland, OH USA

**Keywords:** Aging, Human development, Life-span, Hard-wiring for flexibility, Sociosomatics, Ontogeny, Habitus, Altern, Menschliche Entwicklung, Lebensdauer, Immanente Flexibilität, Soziosomatik, Ontogenese, Habitus

## Abstract

While aging is a general phenomenon that characterizes virtually all species, human aging entails mechanisms of stability and change that comprise distinct or unique features of the species *homo sapiens. *Such mechanisms occur in two interrelated but analytically distinguishable domains, an ontogenetic domain encompassing distinctly flexible structural features of the human organism (world-openness, hard-wiring for flexibility), and the other focused on humans’ uniquely dynamic relation to the environment, encompassing both internalizing processes (habituation) and generative activity (world-construction, imagination). Given this dynamic foundation, biological aging is an incomplete process, which requires human actors to create and sustain the life-worlds they inhabit, worlds which comprise the conditions within which their own aging is constituted and reproduced. The result is the manifest empirical reality of marked cultural and historical variations in human patterns of aging. This means that those who seek to understand human aging must carefully consider how its constitutive dynamics are embedded in culturally and historically specific contexts.

## Abstract (de)

Auch wenn Altern ein generelles Phänomen ist, das nahezu alle Spezies charakterisiert, beinhaltet *menschliches* Altern Mechanismen von Stabilität und Veränderung, die hochspezifisch für die Gattung *Homo sapiens* sind. Diese Mechanismen finden in zwei miteinander verwandten, aber analytisch zu trennenden Bereichen statt: einem ontogenetischen Bereich mit flexiblen strukturellen Merkmalen des menschlichen Organismus (Weltoffenheit, immanente Flexibilität) und einem Bereich, der die spezifisch humane dynamische Beziehung mit der Umwelt umfasst mit internalisierenden Prozessen (Habituation) und generativer Aktivität (Weltkonstruktion, Imagination). Davon ausgehend stellt biologisches Altern einen unvollständigen Vorgang dar, der aktiv Handelnde benötigt, die ihre Lebenswelten schaffen und aufrechterhalten, also Welten, die erst die Voraussetzungen dafür schaffen, dass sich Altern konstituieren und reproduzieren kann. Das Ergebnis ist die offensichtliche empirische Tatsache, dass es deutliche kulturelle und historische Unterschiede in Mustern humanen Alterns gibt. Das bedeutet, dass das Bestreben danach, Altern zu verstehen, diese in kulturelle und historische Dynamiken eingebetteten konstitutiven Dynamiken sorgfältig beachten sollte.

## Introduction: aging and human aging

The phenomenon of age appears as a characteristic of virtually all complex organisms spanning both botanical and zoological domains. Whether aging is viewed as beginning at an embryonic stage of life or at some later point, it represents a near-universal phenomenon across species. For many animals, this generally applies whether the focus is on ontogenetically programmed sequential changes (e.g., fecundity), on molecular damage or other biochemical processes (e.g. shortening/breaking of telomeres; keratinization of ocular gland ducts), on mechanical wear and tear changes (e.g. wearing teeth and joints; cumulative exposure to sunlight) or other factors. Such processes occur while debates continue in areas such as the relative importance of purely temporal processes vs. counts of cell division (e.g. [1]) and the role of factors such as immune responses and diet [2, 3].

While many of these concerns and issues relate to processes of aging across the life world, in this paper we focus on age and aging specifically in humans, which embody key distinctive features compared to the aging and developmental processes of other species. Because much of gerontological science does little to recognize this and assumes continuities between the study of other forms of life and the human sciences, we begin by examining the clear evidence for human distinctiveness.

### Evidence of human distinctiveness

Despite sharing much of our genome and organismic features with a multitude of living genus and species, from beetles to coyotes, human aging is distinctive and in some key ways unique, even among primates. It is marked by patterns not seen and processes not experienced in the lives of other species.

These processes occur through the continuous operation and interaction of at least two broad sets of dynamics, one premised on phenotypic openness of human organisms to contextual forces and the second on human capacity for and indeed necessity of generating social order, and thereby of generating the worlds we inhabit. The dynamic interplay of these two conditions provides the ground for what Berger and Luckmann [4] refer to as the “world-openness” of the human organism, and reflect what Rogoff describes as “hard-wiring for flexibility” [5]. As Sapolsky observed about humans, “the nature of our nature is to be not particularly constrained by our nature” ([6]; see also [7]). These conditions of human distinctiveness have been recognized by scholars across the life sciences, from biology and paleontology [8–10] to the behavioral and social sciences [11–14].

Before we explore the developmental basis for phenotypic flexibility in humans, it is important to recognize at the beginning that humans also create, often without intention, the structuring and restructuring of their own ecological contexts (and those of other species as well) and this extends to the phenomenon of aging. The manifestations of this are myriad. As observed by Berger and Luckmann:

“man’s (sic) relationship to his environment is characterized by world-openness. Not only has man succeeded in establishing himself over the greater part of the earth’s surface, his relationship to the surrounding environment is everywhere very imperfectly structured by his own biological constitution. The latter, to be sure, permits man to engage in different activities. But the fact that he continued to live a nomadic existence in one place and turned to agriculture in another cannot be explained in terms of biological processes” [4, p. 47].

To these observations may be added the point that every human society has a history and undergoes some degree of historical change. This represents a radical departure from the nature of other species who are largely guided by relatively fixed, instinct-directed patterns of behavior [4]. Many such species possess remarkably intricate skills (e.g. of nest, hive or dam building) but they are skills provided by instinct rather than by learning and imagination [13, Chap. 3]. A simple example of this is provided by the recent actions of the State of California, USA, that released 75 beavers in a valley with the explicit goal of returning the region to its original marshland conditions that sustain hundreds of other species [15]. Following their capture and relocation, the beavers simply began their instinctual work of survival all over again. One can immediately understand that this is not the approach in human society.

### Human distinctiveness: implications for aging

The implications for physical and other aspects of aging of being a species shaped by history and culture are pervasive in human societies. As a familiar example, consider the epidemiological transition, resulting in an exponential rise of average human life expectancy in the past two centuries. The extraordinary advances in human longevity have resulted from grand social inventions such as urban sewage systems and discoveries like penicillin. The steady and nearly universal increase in the human life expectancy across the globe [16–18] is a testament to the world-building properties of *homo sapiens *that involve imagination, intentionality and knowledge cumulation and that allow us to shape key aspects of human possibility, including the concept and nature of aging itself. Such developments can obviously not be accounted for by behavior patterns originating at the genetic level, nor by anything other than human activity.

Such advances, all the product of human thought and endeavor, spur scientific enthusiasm with some predicting that major advances in the next decade will offer dramatic new possibilities for offsetting, slowing or reversing what have been taken to be normal aspects of aging, advancing these trends even further [19, 20].

Humans act on their contexts, and at the same time contextual factors impact aging and longevity. Such forces are pervasive in human populations. For example, work by Kelley et al. demonstrates the global impact of food and monetary policy on the genesis and rise of the obesity epidemic. First seen mainly in the USA and high-income countries exposed to the western diet [21], the most devastating impacts are now seen in middle-income countries who were subject to fiscal austerity measures and World Monetary Fund conditions that flooded their markets with processed foods [22]. After increases in average life expectancy in middle-income countries beginning in the 1960s, these regions are experiencing a collision of slowing life expectancy gains and rapid rise of chronic disease, which is again rewriting the questions of what is “old” and what is “aging”? Such questions are of course complicated by enduring debates over how to distinguish and categorize “normal aging” as distinct from age-related disease processes such as osteoporosis or arthritis [19, 23].

Such observations compel several lines of questions about the complex and dynamic character of human aging, and the reasons for and consequences of its historically and culturally variable patterning. We focus here on three such primary lines of inquiry, all of which are fundamentally grounded in the unusual ontogeny of the physical human organism. First, what are the characteristics of the human species and of the human organism that allow for such remarkable variation and change in phenotypic development and aging? Second, given those organismic characteristics, what are the mechanisms by which observed diversity and contingency in patterns of human longevity are produced and regulated? What accounts for its stability, and variability? And third, what are the implications of such characteristics of the organism and mechanisms of regulation for a theoretical understanding of human aging, within and across disciplines? Specifically, what are its implications for the traditional assumptions and premises of gerontology as a field, of sociological, psychological and physiological aging? We consider these three questions each in turn, characteristics primarily in Part I (this paper) and mechanisms and implications mainly in the subsequent Part II.

## The distinct developmental features of *homo sapiens*

As a first step in addressing these questions, we discuss the distinctly human features of development and aging that underlie their contingent character. We identify three broad categories of processes: ontogeny, habituation and generative action (see Dannefer and Perlmutter [24]).

### Ontogeny

As for members of every living species, the ontogenetic physical structure and developmental sequencing of the organism is the foundation upon which patterns of aging are built. One key and necessary step is to identify the age-related aspects of developmental change that appear to have a genetic or species-wide basis. Of course, many aspects of the early life course have somewhat age-bound sequential patterns, such as physical body and brain growth, including puberty and associated changes. With maturity and aging, other changes occur universally or near-universally (menopause, bone density loss) but they occur across a much wider and less fixed chronological range. Other age-related changes are not universal, yet have a clear genetic basis, evidenced by intergenerational transmission (e.g., male pattern baldness, graying hair, auricular hypertrichosis). Any comprehensive understanding of aging must recognize such physically anchored factors and their sources and consequences.

Importantly, however, human beings have proven sensitive to their environments in the activation and timing of some of these “hard wired” processes. For example, average age at menarche has varied widely in human history. In earlier periods of known human history, menarche occurred very early (ages 7–10 years), believed to be a trade-off between survival of the human species and low life expectancy [25]. After a high-water mark of puberty during the eighteenth century (ages 14–15 years), age at menarche has decreased globally, with some girls as young as 8 years old now experiencing their first period [25]. While the causes are debated, most scientists agree that relevant factors include a combination of improved nutrition in childhood, increased body fat/obesity in youth, as well as chemical exposures that disrupt endocrine processes [25–29]. Unlike accelerated aging, which is generally understood to be an individual-level process based on stressors and assaults to the body systems over a person’s lifetime, and therefore manifesting substantial heterogeneity, the average decline in age at menarche has been observed globally and across myriad societal contexts (c. f. [30, 31]).

Even though epigenetic mechanisms are not unique to the human species, the rapidly expanding attention to such processes adds important new challenges and promises for understanding the implications of gene-environment (G‑ E) interaction for aging [32–36]. To call attention to a well-known example, Cole, Hawkley, Arevalo and Cacioppo [37] found evidence suggesting that loneliness is a causal variable that directly effects gene expression in ways that impact the immune system. The regulation of gene expression by social as well as ongoing physiological dynamics (associated with diet, exertion and exercise, toxins, etc.) is both a promising and challenging horizon of research.

Recent attention to epigenetic clocks and scientific pursuits to measure the biological age of humans accurately [38] underscore the growing recognition that markers of chronological age become increasingly imprecise as humans become older, as a lifetime of social and environmental inputs and experiences reach to the cellular level of aging processes [39–41]. Accelerated aging, popularized through Geronimus’s concept of weathering [42], involves mechanisms by which repeated stressors and assaults over the course of one’s life cause physiological and biological functions to operate at a more compromised level than would be predicted by one’s chronological age, leading to earlier onset of chronic disease and frailty. In other words, the body systems dysregulate, or “break down” at younger ages due to external and internal stressors on the body (e.g., stress, diet, environmental exposures, discrimination), such that a younger person’s body systems and chronic disease risk may mimic someone who is much older [43]. This framework has largely been used to explain earlier onsets of chronic disease, metabolic syndrome, and frailty in socially disadvantaged populations [42, 44].

Disentangling chronological age from biological age is not a mere scientific exercise. Understanding the ways that our bodies may “speed up” or “slow down” aging processes brings the most distinctive features of human development into focus. These features constitute a developmental foundation that not only allows for but necessitates that human development be a socially interactive process. They entail plasticity [45], which is a foundation for world-openness, which has also been termed *hard-wiring for flexibility* [5, 6]. Sometimes, such characteristics lead to questions about social determinism. What these features facilitate, however, is not a prescriptive, determinative developmental pathway, but the indeterminacy and potential diversity and innovation in patterns and styles of aging. We elaborate on these points in the next section.

#### Indeterminacy of patterns of development and aging

Such features of human development are founded on the anthropological constants of *exterogestation* (a consequential feature of infancy and early childhood) and *neoteny* (operating over the entire life course). These features of *homo sapiens* have been reviewed in detail elsewhere [10, 12–14, 46] and will be given only briefest treatment here. *Exterogestation* refers to the truncated gestation period and physiologically premature birth of full-term human infants, relative to other species. Exiting the womb and entering the world in a still embryonic state [9], body as well as mind develop in interaction with an active and culturally specific social environment. Correspondingly, *neoteny* (or juvenescence) refers to the exceptionally long *n* period of human childhood and maturation, as well as to the fact that human flexibility and capacities for learning and creativity are not coterminous with youth but extend over the entire life course [8, 10, 13, 14].

Some of the strongest testament to the necessity of human community in shaping its members comes from those who have been tragically deprived of human contact – feral children who tragically endure the years of childhood growth without social life [13, 47, 48]. Children subjected to human exclusion lack language and, if not rescued while very young, apparently outgrow the capacity for developing language. Numerous aspects of the physical development of such tragically isolated young people are also markedly different from “socialized” human beings, thereby indicating centrality of the learning not only of language and but also cultural practices for human development and aging, including significant aspects of physical development [13, 49]. This sensitivity to context is also, of course, reflected in the fact that human tastes, aesthetics and in some respects physical development (e.g., laryngeal and voice development, movement [e.g. ballet] capabilities) vary dramatically by culture.

These general aspects of human development and change are reflected in the founding principles of Vygotskian activity theory which, in contrast to standard organismic theories, recognizes that learning precedes development [50–52], rather than the other way around as is envisioned in the classical organismic paradigm of development represented, e.g., in the work of Piaget, Turiel and many others [53].

Brain-scan evidence has confirmed that the behavioral and cognitive limitations associated with ferality are reflected in severely impeded physical brain development [54], an impediment that can be ameliorated if a child is introduced to and included in healthy social interaction during early childhood. Again, this area of research, therapy and education reflects Vygotskian principles by demonstrating early childhood deficits in physical brain growth that result from feral isolation and lack of social interaction and that such effects can be reversed if the deprived child is afforded human contact in time, i.e., in early childhood [48, 49, 55]. While ferality is of course extremely rare, the general principle demonstrated by such cases, that opportunities for learning account for variation in cognitive development and aging, operates pervasively in everyday experience and social life, as Vygotsky also makes clear.

*Neoteny*, or “juvenescence”, entails an extension of capabilities for learning and growth into adulthood. If “human babies are embryos” [9] it can also be said that human adults are like the children of other species [11–14]. It is increasingly recognized that even in adulthood, learning can drive physical brain growth. This is well illustrated by the research of Maguire et al. [56] on the learning of London taxi drivers. Becoming a licensed taxi driver in London has long required learning maps covering about 25,000 streets (known as *The Knowledge)* and passing a rigorous examination. Following up on their earlier study that found cross-sectional correlations between hippocampus size and “Fhe Knowledge”, Maguire and Woollett [57] conducted a longitudinal study comparing three groups: those who studied and successfully passed the examination, those who studied but did not pass the examination and a control group of those who never studied or took the examination. The research findings showed that trainees who qualified after their training manifested an increase in gray matter volume of posterior hippocampi and memory profile. In contrast, no increase in the gray matter volume of posterior hippocampi was observed among trainees who studied but failed to qualify and the control participants. These powerful findings demonstrate the phenotypic openness that we defined above, specifically that rigorous learning in adulthood can fundamentally alter brain structure and chemistry.

In considering the centrality of interaction and learning in human species-being, one additional and related aspect noted above also warrants added consideration: *homo sapiens* is unique in having constructed habitats over the entire reach of the globe, and potentially even beyond it. Humans do not merely “adapt” to environments; we collectively create the environments we inhabit, both physically and socially [13, 58]. Of course, the diversity of ecologies and the attendant resources and risks are part of what accounts for cross-cultural variations in life patterns and life expectancy noted earlier. One interesting illustration of this point is the extremely unique diet of Sardinian centenarians. Long-touted for being a “Blue Zone” that associates increased longevity with the Mediterranean diet, red wine, and close social ties, more recent evidence among the oldest-old in Sardinia demonstrates culturally unique dietary practices, borne out of famine earlier in the twentieth century, that incorporate unusual ingredients such as clay, heavy minerals, and insects into their diet [59]. In other words, the intersection of human ingenuity to survive and the locally available resources created, perhaps unintentionally, a pocket of extreme longevity.

In sum, human aging cannot be reduced to the internal ontogeny of preprogrammed changes that typify the lives of other species or to general wear factors. While ontogenetic factors are operative, equally integral to human aging are the interactive processes of “mind in society” [52], which account for much of the variation in its forms and patterns. While certain characteristics clearly have a hard-wired ontogenetic foundation, so does the inherent malleability and responsiveness that both enables and requires humans to learn through interaction “hard-wired for flexibility”.

### Habituation

Habituation as a general process is closely allied with similar terms and processes that have been discussed across psychological, sociological and gerontological contexts. It has also been proposed as a central factor to understanding human aging [24]. Of particular note is Kastenbaum’s [60] provocative proposal that aging itself be fundamentally redefined as *hyper*habituation—being stuck in a repetitive world of sameness, with little experience of novel stimulation, challenge or variety. Habituation can be technically defined as “reduction of attention to the familiar”, and Kastenbaum proposes that when the routines and experiences of everyday life are almost entirely contained within “the familiar”, the adverse consequences are themselves constitutive of aging. Kastenbaum argued that hyperhabituation is reflected in familiar associations of aging and old age with boredom, lack of stimulation, and limited opportunities or inclination to embrace novel experiences.

While Kastenbaum’s analysis of hyperhabituation points compellingly to the role of experience in shaping aging, it must be placed in the context of a more general understanding of the construct of habituation. At its most generic level, habituation is a basic organismic learning process [24, 61, 62] which entails developing a response pattern or viable reaction to the range of regularly recurring stimuli an organism encounters. Although this can be seen as a general process of living systems, it is especially crucial for a species that is hard-wired for flexibility and that lacks instinctual or other internal guidance to structure activities, as is the case for *homo sapiens.*

As a concept, habituation has synergies with key principles of general sociological theory. Berger and Luckmann describe a parallel, socially engaged process of habitualization, the process whereby “any action that is repeated frequently becomes cast into a pattern, which can then be reproduced with an economy of effort and which … is apprehended by its performer as that pattern” [4]. Both habituation and habitualization are foundational processes underlying the central Bourdieuian concept of habitus [63]. Habitus is defined as “the way in which the sociosymbolic structures of society become deposited inside persons in the form of lasting dispositions, or trained capacities and patterned propensities to think, feel and act in determinate ways, which in turn guide them in their creative responses to the constraints and solicitations of their extant milieu” [64]. It is the metaphoric ocean of socially organized interactions and experiences that comprise everyday life, “the waters we swim” [65].

#### Hard-wiring for flexibility and processes of habituation

The physiological and developmental bases of “hard-wiring for flexibility” mean that the structuring of routines by repeated and predictable patterns of action, habits, is necessary to guide human activity. Lacking the preprogrammed and instinct-driven life routines and survival patterns that characterize many other species, members of *homo sapiens *must rely on the  processing of information to form plans of action, which from the beginning are for each neonate learned from and then coordinated with others, and mediated through language, arguably the most fundamental social institution.

Thus, from the beginning, human aging and development are phenomena that are culturally and linguistically constituted in a sociohistorically specific set of systemic social networks. The physiological and developmental flexibility and “world-openness” [4] with which each human individual is born ensure that physical as well as cognitive and affective aspects of development are structured by the orderliness of social experience.

The degree of flexibility inherent in processes of human development can be seen in some of the extraordinary aspects of physical development manifested in feral children (e.g., tolerance of extreme cold, quadruped ambulation, unique digit development [49]) but flexibility is much more commonly visible by considering readily observable cultural differences. It is notable, in this respect that both Bourdieu (re. habitus), and Berger and Luckmann (re. habitualization), refer back to Marcel Mauss’s important essay “Techniques of the body” [66]. In this prescient essay, Mauss begins to lay out an age-specific catalog of embodied cultural practices with decisive consequences. Typically, the practices he observed are not intended to respond to age-related issues, indeed they may seem not to be “intended” at all, because they are so pervasively habituated and taken for granted. In his section “Variations of techniques of the body with age”, Mauss shares an observation based on his own wartime experience that well illustrates how social practices can impact development and aging:

“The child normally squats. We no longer know how to… An example: I lived at the front with Australians (whites). They had one considerable advantage over me. When we made a stop in mud or water, they could sit down on their heels to rest, and the ‘sottel’, as it was called, stayed below their heels. I was forced to stay standing up in my boots with my whole foot in the water. The squatting position is, in my opinion, an interesting one that could be preserved in a child. It is a very stupid mistake to take it away from him. All mankind, excepting only our societies, has so preserved it”.

This episode illustrates the perennial but largely invisible phenomenon of *sociosomatics* [4, 13] by which the body is shaped through cultural practices, creating an outcome that is “acquired yet entrenched” [64].

Such acquired but entrenched practices obviously involve a person-environment interaction: they are imposed or provided by society and yet their effects are experienced by and manifested within individuals’ bodies, minds and sense of personhood in ways that structure the physical parameters of aging. While such observations as Mauss offers are rarely concerned with aging, it may be instructive to consider how, e.g., retaining the ability to squat may have implications both for how individuals age, and for what is considered within a given society as “normal aging”.

#### Habitus, sociosomatics and physiological change

As Mauss’s example illustrates, habitus reaches deeply “under the skin”. It entails embodiment of cultural practices and social circumstances, with significant implications for aging and age-related change extending across a wide range of physical characteristics. It is becoming increasingly recognized that such embodiment extends well beyond overt practices and performance to basic physiological functioning. For example, clear cultural differences exist in the relation between age and blood pressure. It is well established that blood pressure increases with age in western industrialized countries [67–69] but in dozens of indigenous societies, blood pressure does not increase [68, 70], cross-context variation often attributed to dietary and lifestyle stress factors. While studies of remote societies are often complicated by ongoing social change as well as by standard methodological issues such as nonresponse, this has been an impressively consistent finding across indigenous populations on multiple continents [68]. As examples, Truswell et al. [71] found that blood pressure did not increase with age among Kung in Botswana. Blood pressure does not appear to increase with age among Yanomano people in Venezuela [72] nor in certain tribes in Brazil [68]. Even when an increase is reported, dramatic variability has been observed in rates of increase. For example, Tsimane adults’ (in Bolivian Amazons) level of systolic blood pressure did increase from age 20 to 70 years but only modestly, 16% for women and only 3% for men.

In the West, the robust finding that blood pressure increases with age is typically treated as “normal”, a taken for granted element of the human condition. Yet it is also known to be associated with modern lifestyle practices, including smoking, obesity, lack of physical activity, high intake of fatty diet, heavy alcohol consumption, low potassium intake and high salt intake. But here, too, evidence from Scandinavia and elsewhere suggests that individuals or communities who engage in alternative, healthier dietary practices show less increase. It is not without reason that some have suggested hypertension is a “disease of civilization” [68].

The key takeaway of this documented variation in “aging” effects on the body is that the impact of social forces is readily manifest not only among those of diverse cultures, but even among those who share the same habitus, albeit with different social circumstances. We have earlier referenced experimental evidence that rigorous study and memorization contribute to physical brain growth, even in adulthood, and it is now robustly established that increases in education reduce the risk of dementia within a given context [73–76]. Evidence also indicates that adults who engage in physical activities, sports and regular exercise in midlife tend to have lower risk of dementia later in life [77–80].

One place where we see evidence of how contextual and social forces shape processes widely associated with biological aging is from studies of gene expression and biomarkers. For example, age-related declines in immune function, immunosenescence, is widely assumed to be a normative factor in human aging [75, 81]. Yet scholars increasingly recognize that quality of immune function has an experiential/social component. As noted earlier, evidence indicates that the experience of loneliness among older adults is linked to the production of leukocytes [82–84]. Those older adults with a high degree of loneliness express 23 different biomarkers differently, all related to suppressed immune system response, greater inflammation, and slower speed of cell replication compared to their non-lonely peers.

Taken together, such findings provide an indication of established and ongoing discoveries of the range and scope of how both social practices and the health consequences of social practices are embodied, resulting in the production of an array of age-related changes. It is telling that Mauss begins his exploration of “techniques of the body” by noting that examples of the sociosomatics he is describing have often been categorized under the heading of “miscellaneous” [66]. Similarly, the disparate examples that we have noted in the foregoing discussion might appear as something of a wide congeries of developmental and age-related physical conditions. Despite their rich and varied manifestations, it is important to recognize that such effects are anything but a miscellaneous congeries. While the outcomes are complex and multi-leveled, they reflect the embodiment of systemic cultural practices, largely habituated into the daily practices of individuals. Despite their strong predictive power, these cultural and social arrangements have often been unnoticed or avoided by the paradigmatic logic of organismic normality that has guided much of our theorizing.

### Generative human action

So far, the discussion has focused largely on how forces external to conscious intentionality or action have an impact on aging, whether from within the body itself (ontogeny) or external to it (habitualization/habituation/habitus). We have not yet considered what is often called the “agentic” role of the actor in producing patterns of aging. At the most general level, it can be said that the routinized flow of everyday life is constantly providing inputs to the organism that influence aging (e.g., through the intake of certain foods, habitual exercise routines that tone muscles or the lack of such exercise, exposure to sun or toxins, etc.).

Yet it can also be observed that in the human case, examples also abound of deliberate efforts of human beings to influence their own aging. The current activities of anti-aging and pro-longevity advocates are well known within and beyond the academy [3, 85–87]. Such advances, all the product of human thought and endeavor, spur scientific enthusiasm with some predicting that major advances in the next decade will offer dramatic new possibilities for slowing or reversing biological aspects of aging, advancing these trends even further [19, 20].

More generally, humans are the only known species to attempt, actively and deliberately, to slow aging and to extend life span. Obviously, many other species, e.g., *C elegans* (the earthworm), tortoises, and hibernating mammals, all have automatic cellular level processes that suspend or slow the aging process for periods of time to enhance survival [88], but these are regulated by instinct and seasonality, not purposeful intention. The earthworm enters into suspended animation in winter and does not age again until the soil warms [89]. Hibernating mammals have cyclical metabolic systems with slower rates of cell division during their period of inactivity, to only that which is necessary to stay alive and then transition into “hyper-drive” during the warm season [90]. Obviously, we have no indications that any of these species enact their routines with a deliberate plan to slow or regulate their aging.

The life expectancy of other species may manifest variation and alteration in response to selective pressures that advantage quality or quantity strategies of survival (as for example in the case of slower aging opossums in predator-free environments [12, 91]); however, the process of natural selection occurred over tens of thousands of generations, and involved no intentional effort on the part of actual opossums.

While the dimensions of intentionality and agentic action as general life-constructing processes have received remarkably little attention in developmental and gerontological discourse, exceptions can be found. A notable one is Lerner and Busch-Rossnagel’s notion of “individuals as producers of their own development” [92–94]. To think of “individuals as producers of their own development” is a beginning, but to leave things at that would be distorted and seriously incomplete.

That is because what is produced through intentional human activity is much more than individual development; it is also the social relations and institutions that comprise the social world, the “waters we swim”, the habitus, the milieu in which individual human actors develop and age [65]. It is only through human’s productive activity that social relationships and hence the social world is generated, including the institutionalized sets of practices ([4, 13, 63, 64, 95]; see also [58]) and the stock of social and cultural knowledge [4] that, among other things, impact aging. Such knowledge and practices comprise precisely the content of the social world that is cognitively internalized and physically embodied through habitualizing processes, in a continuous dialectic of self-society interaction.

It is a mark of the power of the institutions thus created that most of the intentional activity that occurs operates as both social reproduction (or more precisely, reconstitution [96]) and self-reproduction goes largely unnoticed. At least under relative stable and orderly social conditions, the great lion’s share of individual activity simply reproduces the social patterns and individual routines of the prior day. Like many foundational aspects of human experience, the pervasive sweep of this dialectic of self and social reality production is both necessary and essential to human species-being (by providing coherence and predictability to human routines and a context for learning and knowledge cumulation) at the same time that it often also can have obvious adverse effects to the extent that it establishes cultural arrangements that are unhealthy for individuals, and especially so when such practices are so habituated and accepted that they are unquestioned in spite of their harmfulness.

## Conclusion

The evidence marshalled above, drawn from multiple disciplines, makes clear that the empirical character of the aging and age-related change in *homo sapiens* is characterized by a number of key and distinct or even unique attributes. These distinctive features are both structural and developmental, and they entail ontogenetically anchored processes of change as well as, unavoidably, those that have their source in human consciousness, imagination and relationship-building.

In both their healthy and harmful aspects, the cumulative dynamic of such practices and their effects is inherently a temporal set of processes and to the extent that they endure over an extended time period they will inevitably contribute to processes and patterns of individual aging. These processes thus form the distinctive basis of ongoing human practices of self and social reproduction that extend over time and are integral to aging. In the second part of this treatment of human aging, we turn to an examination of the implications for individuals.

## Data Availability

All data supporting the findings of this work are included in the article.

## Declarations

For this article no studies with human participants or animals were performed by any of the authors. All studies mentioned were in accordance with the ethical standards indicated in each case.
